# Hyperpyrexia

**Published:** 1907-11-30

**Authors:** 


### Diseases of Children.

### HYPERPYREXIA.

In children the occurrence of hyperpyrexia during an attack of acute rheumatism is an extremely rare event. At the meeting of the Society for the Study of Disease in Children on November 15, Dr. G. H. Lock reported a fatal case, in a girl aged six years. The temperature rose to 110° F., and was little affected by the treatment adopted. The cold pack was very badly borne.

Hyperpyrexia may occur in other conditions—notably sepsis in the new-born; towards the termination of meningitis, generally of the tuberculous variety; as the result of heat stroke; in malaria; and sometimes at the onset or in the course of malignant fevers. It must not be forgotten that the heat-regulating centres in childhood, especially in infancy, have not acquired the stability of later life, and that irregularities of temperature are produced by much more trivial causes. Mere fright may send a baby's temperature up three or four degrees. Another important point is the fact that an abnormally high temperature may be due to the action of external heat, provided that the means of elimination are restricted. A child's temperature may be raised by the application of hot bottles or by a continuous hot pack. So, too, fever may be maintained by protecting the child from the loss of heat which takes place chiefly from the skin by radiation, evaporation, and conduction.

### Causation.

The explanation of hyperpyrexia in rheumatic fever is a matter of uncertainty. In adults the high fever is sometimes associated with pericarditis. It has been ascribed to the effect of the rheumatic poison on the brain, meninges, or heat-regulating centre. There is no reliable evidence of meningitis in fatal cases, and meningitis of itself does not appear to be the cause of excessively high temperatures. The abnormal rise which often precedes death in tuberculous meningitis, and less frequently in other varieties, is more probably due to destruction of the inhibitory powers of the higher central nervous system on the heat-regulating centre in the medulla than to the actual meningitis.

That toxæmia may be the cause is supported by the occurrence of hyperpyrexia in the virulent cases of toxæmia which are sometimes seen in epidemics of specific fevers and in cerebro-spinal meningitis. In addition to the virulence of the toxin, we have to consider the reaction of the host, a reaction which is more powerful in the young than in older people. Hence, in the case of rheumatic hyperpyrexia in a child we may ascribe the height of the temperature to the virulence of the poison, the instability of the heat-regulating centre, and the deficient resisting power of the body. Another point must be mentioned—namely, decreased loss of heat by evaporation from the skin. In Dr. Lock's case this was especially noticeable, for the skin was dry; and, in fact, high temperatures in rheumatic children are frequently associated with deficient loss of heat by sweating. The characteristic acid sweats of adults are comparatively infrequent in young children.

All the above features of hyperpyrexia suggest that it should occur more frequently in the young than in the old, except in so far as that the loss of heat from the surface of the body is greater in the young than in the full-grown, in the small than in the large animal, according to the relatively greater amount of body surface in proportion to body weight. One is therefore driven to the view that the infrequency of rheumatic hyperpyrexia in the young is due to insusceptibility to the rheumatic poison, or, more probably, that the poison is not developed in any quantity because of their insusceptibility to rheumatic arthritis, the synovial membrane being consequently regarded as the site of its chief development.

### Treatment.

It is essential to reduce fever in these cases. Reduction of temperature does not necessarily shorten disease, but the patient may die from the excessive fever alone. The measures to be adopted vary with the height of the fever, its duration, the nature of the disease, and its probable course. The following methods of treatment must be relied on:—

Eliminate by purgation, lavage, rectal irrigation, diaphoretics and diuretics.

Reduce the sources of heat by putting the patient on a limited fluid diet.

Encourage the loss of heat from the surface of the body by free ventilation of the room, light clothing, and dry atmosphere.

Hydrotherapeutics.—Apply ice or Leiter's coils to the head, or let it rest on a rubber bottle full of cold water, if there are cerebral symptoms. Ice suppositories and rectal irrigations with water at a temperature of 95° F. are useful for infants, if there is not much prostration. Cradling is essential. Sponging should be adopted in all cases in which the temperature is over 104° F., using tepid water, vinegar, or eau de Cologne and water, etc. Or recourse may be had, in more severe cases, to cold fomentations, rubbing the surface of the body with ice, cold packing, or the cold bath. The latter two methods of treatment are often unsuitable to children and may cause shivering, cyanosis, and collapse. It is possible that the temporary application of heat to the skin may be beneficial by inducing dilatation of the constricted cutaneous vessels.

Antipyretic drugs are sometimes necessary, notably when the attendants are stupid or inefficient, and proper nursing is not available. They are most valuable in the high fever of rheumatism, broncho-pneumonia, and enteric fever, but must be used with caution. A combination of phenacetin and caffein citrate is the best, and is safer than antipyrin or acetanilide. It should be given in small frequent doses. Aconite, quinine, and salicylates are also valuable drugs. In some instances nothing produces any sufficient effect.

